# Going full circle: dynamic covalent enzyme immobilisation *via* visually trackable boronate esters

**DOI:** 10.1039/d5sc08585c

**Published:** 2026-03-03

**Authors:** Glenn Bojanov, Juliette Swit, Francesca Paradisi

**Affiliations:** a Department of Chemistry, Biochemistry and Pharmaceutical Sciences, University of Bern Freiestrasse 3 3012 Bern Switzerland francesca.paradisi@unibe.ch

## Abstract

Enzyme immobilisation on solid supports enables biocatalyst recycling but generates significant waste due to single-use resins that are discarded when enzyme activity declines. Here we report a reversible immobilisation strategy based on boronate ester formation between alizarin-functionalised enzymes and boronic acid-modified supports. Alizarin-methyliminodiacetic acid (alizarin-IDA) serves dual roles as both a pH-responsive binding handle and a visual reporter, enabling real-time colourimetric tracking of enzyme loading (red solution → orange resin), immobilisation completeness, and pH-triggered release. A universal labelling protocol was established and successfully applied to four structurally diverse enzymes retaining 77–95% of native activity. All alizarin-labelled enzymes achieved >90% immobilisation yield on different supports, were extensively reusable, and could be removed by acidic treatment with full regeneration of the supports. The load–use–cleave sequence was repeated five times without loss of binding capacity, enabling more than 50 catalytic cycles per support across multiple enzyme lifecycles (5 regeneration cycles × 10+ reactions each) with identical performance.

## Abstract 2

Alizarin-IDA as binding handle and colourimetric process reporter for reversible boronate-ester immobilisation of structurally diverse enzymes. Supports are fully regenerated across 5 enzyme lifecycles, delivering 50+ catalytic cycles per carrier.

## Graphical abstract

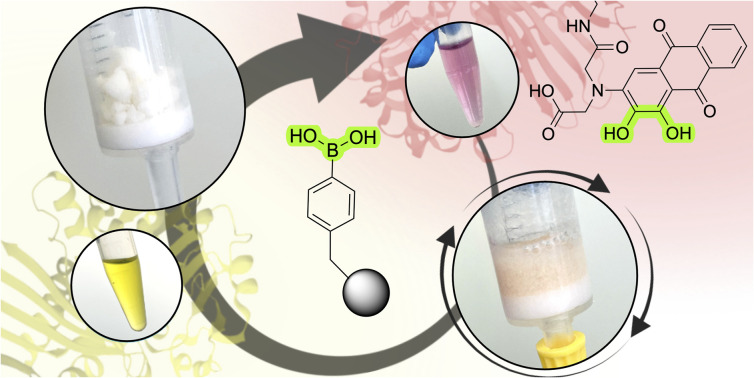

## Introduction

Biocatalysis delivers high chemo-, regio-, and stereoselectivity under mild conditions and is widely deployed across fine chemicals, pharmaceuticals, food, and fuels.^1–3^ In practice, these processes often depend on immobilised enzymes for recovery and process integration, yet implementation remains materially linear: carriers, which constitute ∼90–99% of the immobilised catalyst mass, are discarded when the enzyme deactivates.^4–6^ Lipase B from *Candida antarctica* (CALB) has been one of the first immobilised lipases *via* non-covalent adsorption on an acrylic polymeric resin. Commercialised as Novozym 435 by Novonesis, it is likely the most used biocatalyst in academia and industry.^7^ To prevent enzyme leaching and maximise stability, irreversible covalent bonds are favoured in industry,^8–10^ this however creates a dilemma: the same chemistry that enables robust catalysis prevents carrier regeneration. From a circular economy perspective, extending the lifetime of the support, not only of the enzyme, is a direct but underused lever to reduce cost and environmental footprint.^11–13^ The rationale for immobilisation over the use of soluble enzymes has been extensively discussed in terms of ease of separation (downstream processing), operational stability, and reactor integration.^14,15^ Aside from non-covalent chemistries which easily enable enzyme desorption, dynamic covalent chemistries are less explored in this field but are well established in bioconjugation for drug-delivery where the payload must be released under controlled but mild conditions.^16,17^ Imine, oxime, and hydrazone chemistries enable reversible bioconjugation, but their hydrolytic lability under typical reaction conditions would not make them suitable for enzyme immobilisation in biocatalytic processes.^18,19^ Disulfide exchange has demonstrated promise for reversible enzyme attachment, but introduces redox sensitivity that complicates processes involving reducing agents or oxidizing substrates.^20–22^ Boronate-ester formation between boronic acids and diols offers a compelling alternative. The reaction proceeds rapidly and reversibly in water: catechols exhibit high affinity at pH 7–9 and are released upon acidification or in the presence of competing polyols.^23–26^

Boronate-affinity chromatography leverages this principle for the purification and separation of glycoproteins bearing native *cis*-diol carbohydrates.^27–29^ Several studies have applied this chemistry to protein immobilisation for sensing, but remain limited to naturally glycosylated proteins.^30,31^ One study broadened the scope beyond glycoproteins by successfully immobilising penicillin G acylase from *E. coli*, a non-glycosylated enzyme, on phenylboronate-activated supports.^32^ Multipoint attachment yielded stabilisation in organic solvents, and reversible desorption with mannitol was demonstrated, though support regeneration for repeated biocatalytic use was not explored. Beyond protein applications, the pH-responsive reversibility of boronate esters has been well-established in drug delivery systems,^33–35^ demonstrating pH-triggered release under mild conditions.

Despite these precedents, no method exists for biocatalysis that: (i) installs boronate-reactive handles on any enzyme regardless of native glycosylation, (ii) enables visual tracking of immobilisation and release, and (iii) permits true carrier regeneration across multiple enzyme lifecycles. Here we combine function and readout in a single molecular handle to enable cradle-to-cradle enzyme immobilisation. Phenylboronic-acid groups are installed on the carrier (chemically robust, batch-prepared, storable) and enzymes are labelled through surface lysine amino acids with alizarin-3-methyliminodiacetic acid (alizarin-IDA), which supplies both a catechol for boronate-ester formation and a vivid, pH-sensitive chromophore.^36–39^ The resulting red (free) → orange (bound) → yellow (released) colour sequence provides direct visual verification of each process step.^24,36^ This approach aims to establish a general immobilisation strategy compatible with diverse enzymes classes, standard support materials, and packed-bed operation, offering a practical route towards support regeneration in industrial biocatalysis.^40–44^

## Results and discussion

**Fig. 1:**
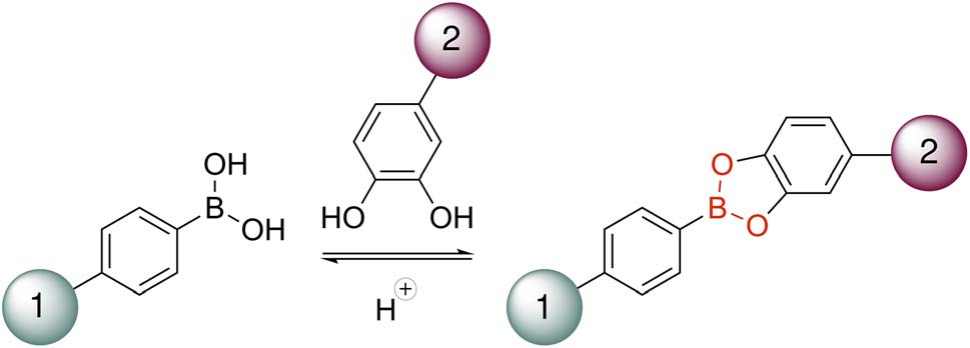
Reaction scheme reversible boronic ester formation and cleavage. (Case a) 1 = enzyme and 2 = resin. (Case b) 1 = resin and 2 = enzyme.

To evaluate our immobilisation strategies, we selected a panel of five structurally diverse enzymes representing different fold families and oligomeric states: *Mycobacterium smegmatis* acyltransferase (*Ms*AcT, octamer), *Candida boidinii* formate dehydrogenase (*Cb*FDH, dimer), *Streptomyces pristinaespiralis*l-lysine cyclodeaminase (*Sp*LCD, tetramer), *Thermomyces stellatus* (*R*)-amine transaminase (*Ts*RTA, dimer), and commercial porcine trypsin (monomer). Our initial screening explored the derivatisation of enzymes surface lysines with boronic acid in order to introduce the acidic binding handle while a set of resins were modified with dopamine to provide the catechol moiety for the reversible covalent interaction (Fig. 1, case a). Two structurally distinct enzymes were modified with 4-formylphenylboronic acid (FPBA): *Ms*AcT, a benchmark transesterification catalyst, and *Cb*FDH, essential for NAD^+^ regeneration. The number of exposed surface lysines on each enzyme was estimated from the available crystal structures (28 in *Ms*AcT and 38 in *Cb*FDH). FPBA was then reacted with the enzymes *via* reductive amination in a 1 : 1 molar ratio with respect to the total surface lysines. *Ms*AcT maintained native–level activity (229 U mg^−1^), while *Cb*FDH exhibited a modest increase (3.01 *vs.* 2.68 U mg^−1^). In parallel, two different commercially available resins were functionalised with dopamine to introduce the catechol binding partner. Agarose resin required epichlorohydrin activation followed by acid ring-opening and periodate oxidation to generate aldehydes as previously described.^47^ EP400 epoxy resin was directly converted *via* acid ring-opening and periodate oxidation.^48^ Dopamine (5-fold molar excess) was then conjugated to both glyoxyl resins through reductive amination, introducing the catechol binding partner.

**Fig. 2:**
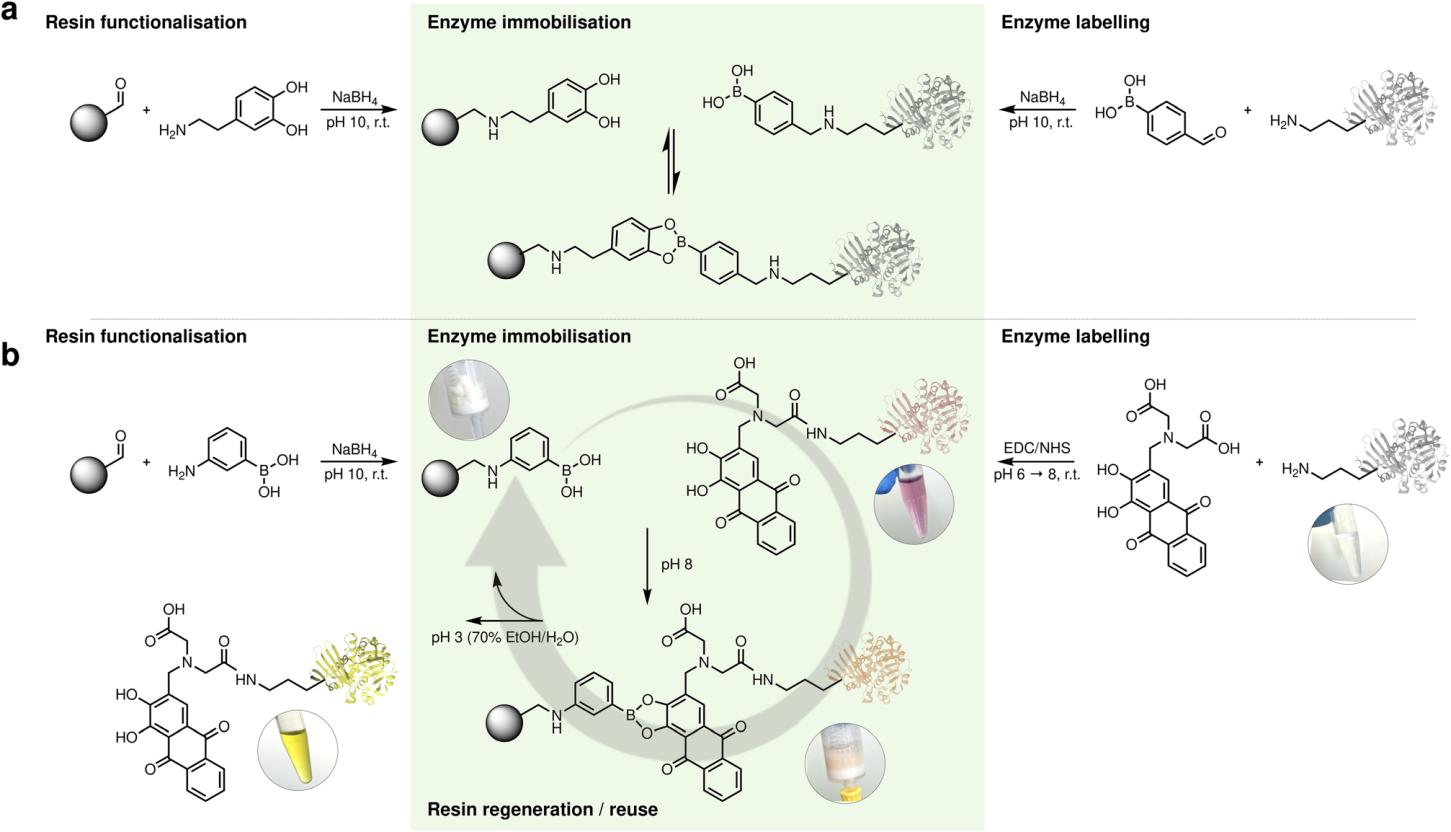
Strategies for reversible enzyme immobilisation through boronate ester formation. (a) First-generation design: dopamine introduced onto aldehyde-activated resins *via* reductive amination (NaBH_4_), followed by binding of boronic-acid-modified enzymes. (b) Inverted design: boronic-acid resins prepared analogously, while enzymes are labelled with alizarin-IDA through EDC/NHS coupling to lysine residues. The resulting catechol–boronate ester provides pH-responsive, visually trackable, and reversible immobilisation.

An overview of the setup is shown in Fig. 2a. Simple incubation of the FPBA-modified enzymes with the dopamine-functionalised resins for 2 hours showed high immobilisation yields (>99%) on both agarose and methacrylate support. However, the recovered activity (RA) was strongly influenced by the FPBA : lysine ratio used during enzyme functionalisation. *Ms*AcT retained 11–14% activity on methacrylate and up to 38% on agarose, substantially lower than the 73% reported for traditional multipoint covalent immobilisation on agarose.^49^ Reducing the FPBA : lysine ratio to 0.7 : 1 improved activity retention showed a similar trend, with RA improving from 30 to 44% on methacrylate as the ratio decreased from 0.5 : 1 to 0.2 : 1. These results demonstrated a clear correlation between labelling density and activity retention, suggesting that excessive surface modification disrupts the native enzyme structure, while moderate functionalisation preserves catalytic function.

Despite the success of our initial protocols, critical limitations emerged with the dopamine-based system. Progressive oxidation of dopamine residues caused resin darkening and compromised binding efficiency, which is particularly problematic for batch-prepared resins requiring long-term storage (Fig. S1). Furthermore, enzyme cleavage proved difficult to monitor: extensive screening of conditions (pH 2.5–4, competing diols, Ca^2+^) yielded no detectable protein in supernatants by Bradford assay, possibly due to protein precipitation under desorption conditions. While through SDS-PAGE analysis of urea-treated resins, proteins cleavage could be at least confirmed, the quantification remained challenging (Fig. S2).

In an effort to simplify the process and enhance chemical stability, we inverted the location of the chemical handles: the stable boronic acid moiety was to be placed on the resin (suitable for batch functionalisation and long-term storage) while the oxidation-prone catechol would be used to derivatise the enzyme, where it would be freshly installed on demand (Fig. 1, case b). This inversion would not only eliminate resin degradation over time but also avoid catechol instability during cleavage, ensuring reliable regeneration and consistent performance across cycles.

**Fig. 3:**
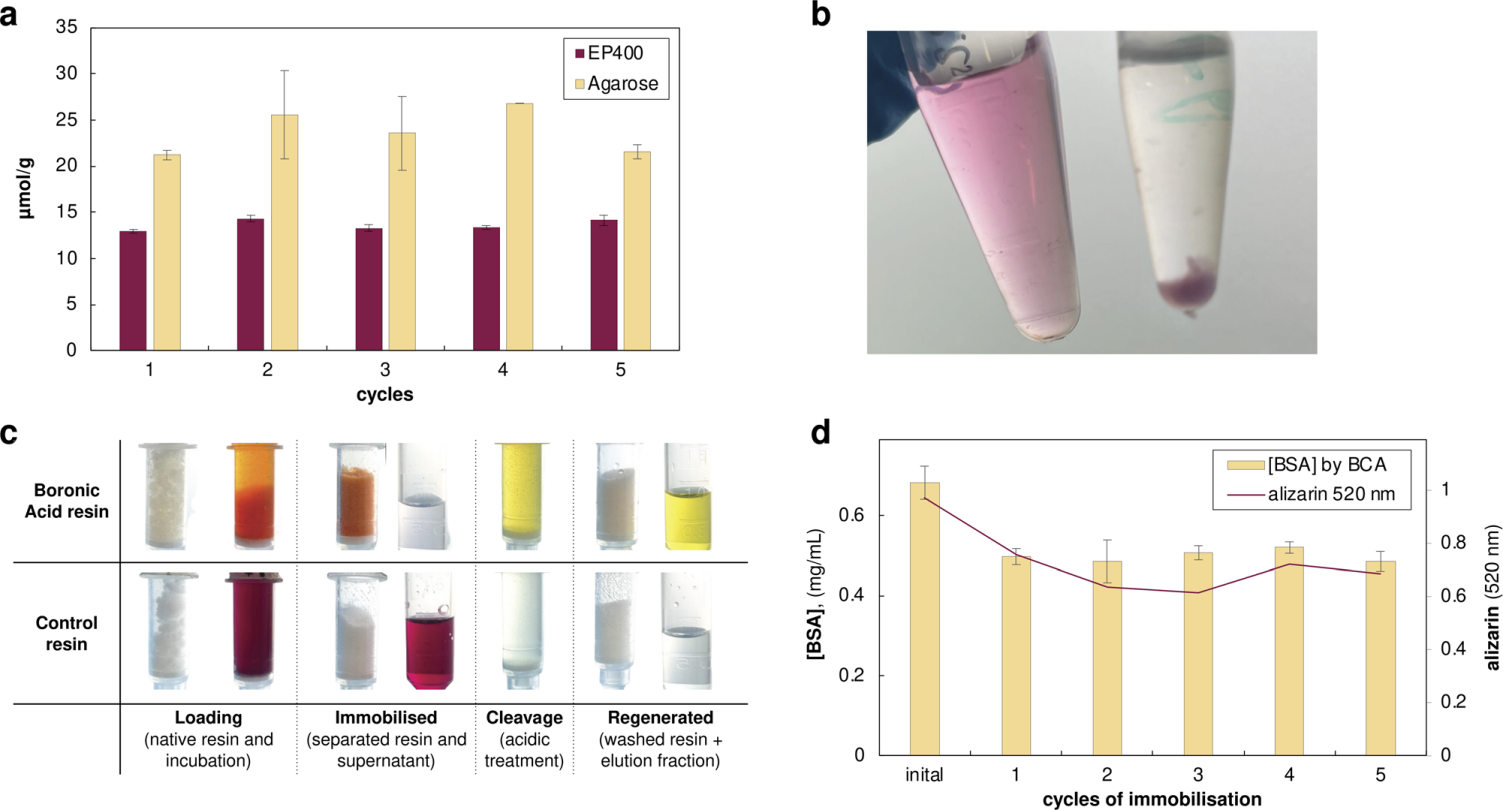
Reversible enzyme immobilisation *via* boronate ester formation. (a) Alizarin binding capacity of EP400 methacrylate and agarose resins over five regeneration cycles. (b) Alizarin-labelled enzyme solutions (left) and thermal denaturation test (right) showing the red protein pellet with a colourless supernatant, confirming the covalent attachment of the dye. (c) Visual tracking of the immobilisation process: alizarin-labelled BSA (red) binds to boronic acid resin forming orange resin with colourless supernatant. Acidic cleavage releases protein as a yellow solution. (d) BSA protein recovery during five immobilisation–cleavage cycles monitored by BCA assay and alizarin absorbance (520 nm); see Fig. S3 for detailed mass balance.

Following the design inversion, we first prepared boronic acid-functionalised EP400 methacrylate and agarose supports. To validate the functionalisation, we used alizarin (1,2-dihydroxyanthraquinone), an established colourimetric probe for boronate ester formation. Quantitative binding experiments revealed functional capacities of 13.6 ± 0.6 µmol alizarin per gram for EP400 resins and 23.7 ± 2.0 µmol g^−1^ for agarose supports, corresponding to the boronic acid groups on the support available for enzyme immobilisation (Fig. 3a). Control experiments with non-functionalised resins showed negligible alizarin binding (<0.5 µmol g^−1^), confirming the specificity of the boronate–alizarin interaction.

To our delight, the alizarin quantification experiments offered a striking colour variation. The deep red alizarin solution turned bright orange immediately upon contact with boronic acid resins, with the resin beads developing intense colouration within seconds, while the surrounding solution rapidly lost its colour (Video S1). After separation, the resins retained the orange colour while supernatants remained colourless. Control native resins showed no colour change, with any slight red hue quickly removed after washing with water. Acidic treatment (70% ethanol/30% water with HCl, pH ∼3) released alizarin quantitatively as a yellow solution, returning the boronic functionalised resin to its original white appearance. The resins maintained full binding capacity over five sequential quantification cycles, demonstrating the robustness of the boronic acid functionalisation (Fig. 3a).

Crucially, in this experiment alizarin served as a small-molecule mimic of the dopamine-functionalised enzymes, demonstrating that catechol–boronate ester formation and cleavage were indeed rapid and efficient, even though no enzyme was present in these initial validation experiments. This distinct colour sequence—red (free) → orange (bound) → yellow (acidic released)—provided unprecedented visual tracking of each process step without analytical instrumentation. This clear visual confirmation of complete catechol release demonstrated effective cleavage, resolving the detection challenges encountered with the dopamine system, where low protein recovery had complicated assessment. This observation suggested that alizarin-based molecules could serve dual roles as both binding handles and integrated visual indicators for process monitoring well beyond their use as a diagnostic probe. Its chemical stability compared to dopamine, combined with the visual clarity demonstrated in our validation experiments, prompted us to explore alizarin derivatives as the enzyme-labelling moiety itself. We identified alizarin-3-methyliminodiacetic acid (alizarin-IDA), which retains the catechol functionality while bearing a carboxylic acid handle suitable for EDC/NHS coupling to surface lysines (Fig. 2b). To evaluate this concept, we first tested alizarin-IDA conjugation to bovine serum albumin (BSA) as a model protein. Following EDC/NHS activation and coupling at pH 8, the reaction mixture was purified using PD-10 desalting columns to remove excess unreacted dye. The eluted BSA fraction displayed a distinct pink-red colouration, again a striking visual change from the typical colourless protein solution. UV-vis spectroscopy showed an absorption maximum at 520 nm, demonstrating alizarin conjugation. To confirm covalent nature of this attachment, we boiled the labelled BSA solution. Upon thermal denaturation, the protein precipitated, leaving a colourless supernatant while the protein pellet remained vividly red (Fig. 3b). The reversibility and visual tracking capabilities were systematically evaluated through five complete immobilisation–cleavage cycles (Fig. 3d). When alizarin-labelled BSA was incubated with one of the boronic acid-functionalised resin (agarose was selected for its higher binding capacity) at typical enzyme loading conditions (5 mg protein per g resin), we observed immediate and quantitative binding: the resin turned bright orange while the supernatant became colourless, with both Pierce™ BCA Protein Assay (Thermo Scientific, Cat. No. 23225) and 520 nm absorbance confirming nearly complete protein immobilisation. Control resins showed little protein binding, verifying the specificity of the boronate–catechol interaction. The covalent boronate ester formation was confirmed through standard thermal challenge experiments. While control experiments with unlabelled BSA showed complete protein release upon boiling, alizarin-BSA remained bound to the resin, which retained its orange colour. Both the flow-through collected after initial loading and the supernatant recovered after boiling the loaded resin showed negligible protein content (<5% of loaded amount) and minimal alizarin absorbance, demonstrating stable attachment under harsh conditions. Only upon acidic treatment did the protein release occur, accompanied by the expected colour transition, with the orange resin turning to white and the elution fraction yellow. Complete protein removal was confirmed when both BCA measurement and alizarin absorbance at 520 nm returned to baseline after washing with 3 resin volumes (fractions were pooled and neutralised before measurement). Subsequent neutralisation and reloading with fresh alizarin-BSA restored the orange resin colouration, confirming the reversibility and reusability of the system for at least 5 cycles (Fig. 3d).

Having established alizarin-IDA as a functional enzyme label, we sought to develop a standard functionalisation protocol applicable across diverse enzyme classes available either in our laboratory or commercially. Initial optimisation studies revealed that reaction parameters (pH, time, temperature, and alizarin-to-lysine ratios) could be fine-tuned for individual enzymes to maximize both labelling efficiency and activity retention. However, the practical implementation of this technology demanded a simpler approach: a single, robust protocol that would work sufficiently well across a broad spectrum of enzymes without requiring case-by-case optimisation.

The challenge lies in the inherent structural diversity of enzymes. Our analysis of several structurally distinct enzymes using both SASA calculations and CapiPy-assisted surface mapping (a software tool designed to rationalise immobilisation strategies) revealed dramatic variations in lysine accessibility.^50^ As shown in Table 1 the number of surface-exposed lysines (SASA ≥ 80 Å^2^) ranged from just 2 in *Sp*LCD to 38 in *Cb*FDH. Moreover, the reactivity of these lysines, which is influenced by local microenvironments, p*K*a variations, and dynamic protein motions, proved difficult to predict *in silico*. This challenge is well-recognised in enzyme immobilisation, where case-by-case empirical optimisation typically remains necessary. We therefore sought to establish a general protocol that could accommodate such structural diversity and serve as a practical starting point for further optimisation. Through experimental optimisation, we established that effective labelling occurred within a range of 1–10 alizarin molecules per reactive surface lysine. To ensure broad applicability, we designed a protocol using a 100-fold molar excess of alizarin-IDA relative to total enzyme concentration (equivalent to a 1 : 100 enzyme : dye ratio and at least 6 times the amount of lysines). This substantial excess ensures that even enzymes with numerous surface lysines achieve adequate labelling within the optimal range, while the mild conditions preserve enzymatic activity. We validated this strategy across a panel of structurally diverse enzymes: *Ms*AcT (octameric acyltransferase), *Cb*FDH (dimeric formate dehydrogenase), *Sp*LCD (tetrameric cyclodeaminase), *Ts*RTA (dimeric transaminase), and commercial trypsin from porcine pancreas (lyophilised powder). These enzymes span different fold families and oligomeric states (monomer to octamer), ranging from 25 to 187 kDa in their assembled forms, representing a broad cross-section of industrial biocatalysts. All enzymes displayed the characteristic pink-red colouration upon labelling, with absorption maxima at 520 nm confirming successful alizarin conjugation. Quantification of the Degree of Labelling (DoL) revealed modification levels ranging from ∼5 alizarin moieties for *Ts*RTA and trypsin to ∼38 for *Cb*FDH, in line with the abundance of surface-accessible lysines (Table 1). These values rationalise the observed differences in enzyme performance: while *Ms*AcT proved remarkably robust despite ∼15 modifications, the saturation of available lysines in *Cb*FDH (∼38 labels) likely restricted conformational flexibility, contributing to its lower recovered activity (43%, Table 2). Conversely, the low activity of trypsin is likely attributed to autolysis during the process rather than specific labelling interference. Critically, enzymatic activity was preserved across the panel. With the exception of trypsin, the enzymes retained 77–95% of their native activity.

**Table 1:** Overview of the structurally diverse enzymes tested for universal alizarin-IDA functionalisation. The table summarises the quaternary structure, molecular weight (MW), calculated number of exposed surface lysines, and the molar ratio of dye to lysine used during the labelling reaction. The final Degree of Labelling (DoL) was determined experimentally by integrating the absorbance spectrum of the purified enzyme (see Fig. S5 for calibration and integration details) and normalising to the protein concentration

Enzyme	Quart. structure	MW (kDa)	Exposed Lys (calc.)	Reaction ratio (dye: Lys)	[Enzyme], mg mL^−1^	Experimental DoL (mol mol^−1^)
*Sp*LCD	Tetramer	149.5	2	50	2.0	6.9
*Cb*FDH	Dimer	161.9	38	6	0.1	38.4
*Ts*RTA	Dimer	82.9	24	8	4.0	4.8
*Ms*AcT	Octamer	187.6	28	28	1.6	15.3
Trypsin	Monomer	25.0	6	16	1.2	5.1

**Table 2:** Enzyme functionalisation using universal protocol: activity retention after labelling, immobilisation yield (IY), and recovered activity (RA) after immobilisation^a^

Enzyme	Post-labelling	Immobilisation			Post-immobilisation
	Activity	Resin	Loading (mg g^−1^)	IY	RA
*Sp*LCD	Traces^b^	Agarose	5	99%	n.d.
*Cb*FDH	77%	Agarose	5	92%	43%
*Ts*RTA	92%	Agarose	5	91%	72%
		EP400	10	93%	48%
*Ms*AcT	95%	Agarose	5	94%	93%
Trypsin	57%	Agarose	5	78%	22%

a Activity is intended as the retained after alizarin-IDA labelling and purification, relative to native enzyme. RA: recovered activity of immobilised enzyme relative to native free enzyme.

b *Sp*LCD activity could not be accurately quantified due to interference between alizarin and the FMOC derivatisation required for product detection by HPLC assay; conversion was clearly observed but not accurately quantifiable.

With our functionalised enzyme panel in hand, we evaluated their immobilisation performance on the boronic acid-modified agarose resin at a typical loading of 5 mg enzyme per g support. All enzymes showed rapid binding behaviour similar to BSA, achieving >90% immobilisation efficiency within 2 h as determined by both colourimetric (at 520 nm) and Bradford analysis of residual supernatants (Table 2). Control experiments with unlabelled enzymes showed minimal (<10%) non-specific adsorption, typical for agarose supports.^51^ The immobilised enzymes retained catalytic activity across the panel, with RAs ranging from 22% (trypsin) to 93% (*Ms*AcT). Notably, *Ms*AcT and *Ts*RTA showed particularly high activity retention, making them suitable candidates for further characterisation. For *Ts*RTA, we extended the boronate ester immobilisation to EP400 methacrylate resin and compared it with the previously optimised covalent epoxy immobilisation performed at higher enzyme loadings (10 mg g^−1^) on the same support.^45,46^ When compared with the optimised conditions, the boronate ester immobilisation on EP400 achieved 93% IY with 48% RA (12.9 ± 2.2 U g^−1^), closely reproducing the performance of the epoxy methacrylate reference (>99% IY and 53% RA 13.7 ± 1.8 U g^−1^). This demonstrates that the reversible boronate ester linkage delivers comparable catalytic performance to irreversible covalent attachment, while enabling support regeneration.

**Fig. 4:**
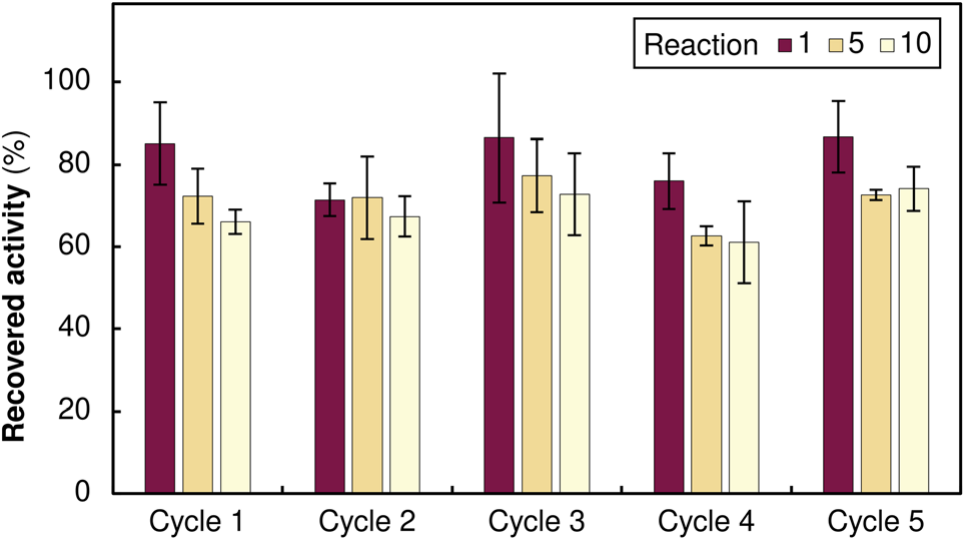
Activity of immobilised alizarin-functionalised MsAcT (relative to the free enzyme) across five complete load–use–cleave–reload cycles. Each cycle comprised 10 consecutive reaction cycles, with activity measured at the 1st, 5th, and 10th reactions, followed by pH-triggered enzyme removal and reloading of fresh enzyme onto the regenerated boronic acid support.

To gain further insight into the binding mechanism, we monitored the immobilisation kinetics using *Ts*RTA as a model system. The process proved extremely rapid, with ∼50% of the protein immobilised within the first minute and >75% binding achieved within 15 minutes (Fig. S6). This rapid binding confirms that boronate ester formation is kinetically efficient, minimising the time required for resin loading. Eventually, to assess the full potential of our system, we performed multiple loading, cleaving, and reloading experiments using *Ms*AcT as a representative biocatalyst. Each immobilisation cycle consisted of: (i) enzyme loading, (ii) 10 repeated reaction cycles, and (iii) enzyme removal and resin regeneration. The immobilised *Ms*AcT maintained stable activity throughout 10 consecutive catalytic cycles, with no significant loss. Following this, the enzyme was removed by acidic wash, the orange-to-white transition confirming complete stripping, and fresh alizarin-functionalised *Ms*AcT was loaded onto the regenerated resin. This complete sequence was systematically repeated 5 times, with identical performance of both the enzyme and the resin. The consistent activity across all immobilisation cycles confirms true cradle-to-cradle operation: each resin support enables 50+ reaction cycles (5 enzyme loadings × 10+ uses each) before any potential disposal (Fig. 4).

Finally, we assessed the pH stability of the boronate–catechol linkage under operationally relevant conditions (Fig. S4A). Alizarin-loaded boronic acid agarose resin was incubated between pH 3–10 for 2 h to assess spontaneous hydrolysis. The complex remained stable across a wide pH range (3–8), with only moderate release at pH 9–10 (6–17%) (Fig. S4A). This alkaline instability likely arises from hydroxide-mediated mechanisms, distinct from the protonation-driven dissociation observed at low pH; however, we selected acidic cleavage for regeneration as it offered superior speed and completeness. Beyond pH, we evaluated stability against common biocatalytic additives (Fig. S4B). Incubation with high ionic strength buffers (300 mM NaCl), reducing agents (1 mM DTT), competing polyols (10% glycerol), or substrates (formate) resulted in negligible protein leaching (<6%). These results confirm that the boronate ester bond is sufficiently stable for biocatalytic operation across the pH range (6–8), where most enzymes maintain high activity,^52^ while still permitting controlled cleavage at very low pH.

## Conclusions

Enzyme immobilisation has long faced a fundamental dilemma: the irreversible covalent bonds that enable robust industrial biocatalysis simultaneously prevent carrier regeneration, generating substantial waste when activity declines. Here we address this through boronate ester chemistry combined with alizarin-IDA, a bifunctional molecule that serves as both a pH-responsive binding handle and as an integrated chromophoric reporter. By incorporating alizarin-IDA directly on the enzyme surface we established a system that enables: (1) direct confirmation of successful enzyme labelling *via* the distinct red colour of functionalised enzyme solutions; (2) visual tracking of IY during resin loading (red → orange shift; transparent supernatant); (3) immediate, reagent-free confirmation of pH-triggered release during cleavage (orange → yellow elution; resin turns colourless): (4) operational transparency across multiple loading-use-cleave cycles without the need for analytical instrumentation.

We established a universal labelling protocol effective across five structurally diverse enzymes (25–187 kDa, monomer to octamer). All alizarin-functionalised enzymes retained 77–95% native activity and achieved >90% immobilisation yields with typical enzyme loadings. Commercial trypsin and *Sp*LCD were the weakest performers which is possibly linked to the limited lysines available for reactivity. Critically, for *Ts*RTA we further showed, that the chemistry extends to methacrylate supports and that the performance matched traditional covalent epoxy immobilisation at higher loadings (10 mg g^−1^). *Ts*RTA retained 48% activity *via* boronate esters *versus* 53% *via* irreversible epoxy chemistry, whilst enabling complete support regeneration.

Multiple cycling with *Ms*AcT could be carried out for at least five complete load–use–cleave sequences, enabling 50+ reaction cycles per support with no loss of performance. This represents a step-change in support utilisation: rather than discarding 90–99% of catalyst mass after a single enzyme lifetime, carriers now serve multiple lifecycles.

In combination with its wide operational pH range, the modular, aqueous chemistry provides broad compatibility with diverse enzymes, buffer systems and process configurations (*i.e.* packed-bed operation and standard process equipment). Looking forward, this dynamic binding opens opportunities beyond traditional support regeneration. The specific pH-triggered release enables enzyme immobilisation on high-value materials, such as electrodes for biosensors, optical fibres for process monitoring, or specialised coatings on medical devices, where recovery and reuse of both the enzyme and the substrate become economically justified. The visual tracking and reversibility are well-suited for automated flow systems with in-line regeneration and real-time process monitoring, whilst 3D-printed scaffolds and microfluidic devices could exploit the on-demand loading capability for spatially programmed biocatalysis. This work provides a practical route to address material circularity in industrial biocatalysis, demonstrating that catalytic performance and sustainability are compatible goals, and that reversibility expands rather than limits the scope of enzyme immobilisation.

## Author contributions

G. B. conceived and developed all experiments on the alizarin-IDA immobilisation strategy, analysed data, and wrote the manuscript. J. S. performed the initial dopamine-based immobilisation experiments. F. P. conceptualised and supervised the project, contributed to experimental design and data interpretation, and co-wrote the manuscript. All authors have read and approved the final version.

## Conflicts of interest

There are no conflicts to declare.

## Supplementary Material

SC-OLF-D5SC08585C-s001

SC-OLF-D5SC08585C-s002

## Data Availability

The data supporting this article have been included as part of the supplementary information (SI). Supplementary information is available. See DOI: https://doi.org/10.1039/d5sc08585c.
