# Supplementary material for: Going full circle: dynamic covalent enzyme immobilisation via visually trackable boronate esters
Source: Chem Sci. 2026 Mar 3;17(17):8513–20. doi: 10.1039/d5sc08585c (PMC12978351; doi:10.1039/d5sc08585c)
Supplement: SC-017-D5SC08585C-s001 [file SC-017-D5SC08585C-s001.pdf]

Supplementary Information (SI) for Chemical Science.
This journal is © The Royal Society of Chemistry 2026

Going full circle: Dynamic covalent enzyme immobilisation via visually trackable boronate esters

Glenn Bojanov, [a] Juliette Swit [a] and Francesca Paradisi *[a]

[a] *Department of Chemistry, Biochemistry and Pharmaceutical Sciences, University of Bern, Freiestrasse 3 3012 Bern, Switzerland.*
** Corresponding author email address: francesca.paradisi@unibe.ch*

Table of Contents

Materials And Methods 3

Protein Expression And Purification 3

Initial Screening: Dopamine-Based Immobilisation System 3

Enzyme Cleavage Under Initial Design Conditions 4

Reversible Immobilisation And Resin Regeneration With Alizarin-Labelled BSA 4

Operational Stability 5

Determination of Degree of Labelling (DoL) 5

Immobilisation Kinetics 6

Experimental 6

References 9

Materials and Methods

General considerations

All chemicals and reagents were obtained from commercial suppliers and used without further purification unless otherwise stated. Agarose 6BCL beads were purchased from ABT Beads (Spain). EP400 epoxy methacrylate resin was obtained from Resindion S.R.L. (Italy). PD-10 desalting columns were purchased from Cytiva (GE Healthcare). All buffers were prepared using ultrapure water (Milli-Q system, Merck) and filtered through 0.22 µm membranes before use.

Instrumentation

Protein concentrations were determined using a NanoDrop spectrophotometer (Thermo Fisher Scientific) at 280 nm using calculated extinction coefficients from ExPASy ProtParam, or by Bradford assay (Bio-Rad) for concentrations below 1 mg/mL. UV-Vis absorption spectra were recorded using an Agilent Cary 8454 UV-Vis spectrophotometer. Enzyme activity assays were performed using a BioTek Synergy H1 microplate reader in 96-well plates at the specified temperatures. HPLC analysis was performed on a Dionex UltiMate 3000 system (Thermo Fisher Scientific). SDS-PAGE analysis was performed using 12% Mini-PROTEAN TGX precast gels (Bio-Rad) and visualized by Coomassie Brilliant Blue staining. Protein purification was conducted using an ÄKTA pure FPLC system (Cytiva). Mini Bio-Spin chromatography columns (Bio-Rad, 1.2 mL bed volume) and 5 mL polypropylene syringes equipped with polyethylene frits were used for immobilized enzyme activity measurements.

Protein Expression and Purification

Table S1: Protein expression and purification conditions. Optimised expression conditions including host strain, temperature, induction method, and duration, along with purification buffer compositions for each enzyme used in this study.

ID	Characteristic	Optimised expression condition	Purification	Extinction coefficient (ε, M^{-1} cm^{-1}, ExPASy)
*Sp*LCD	*Streptomyces pristinaespiralis*	*E. coli* BL21(DE3) Star, LB, OD_{600} 0.6–0.8, 0.1 mM IPTG, 25 °C, 20 h	Ni-NTA, ÄKTA (50 mM KPi, 100 mM NaCl, 10/300 mM imidazole, pH 7.0)	26595
*Cb*FDH	*Candida boidinii*	*E. coli* BL21(DE3) Star, TB, 37 °C, 8 h → 25 °C, 20 h	Ni-NTA, ÄKTA (50 mM KPi, 300 mM NaCl, 30/300 mM imidazole, pH 7.5)	51465
*Ts*RTA	*Thermomyces stellatus*	*E. coli* BL21(DE3) Star, TB, 5 g/L lactose, 37 °C, 4 h → 25 °C, 20 h	Ni-NTA, ÄKTA (50 mM KPi, 100 mM NaCl, 0.1 mM PLP, 10/300 mM imidazole, pH 8.0)	53860
*Ms*AcT	*Mycobacterium smegmatis*	*E. coli* BL21(DE3) Star, LB, 0.5 mM IPTG, 20 °C, 20 h	Ni-NTA, gravity (50 mM KPi, 100 mM NaCl, 10/300 mM imidazole, pH 8.0)	28085

Initial Screening: Dopamine-Based Immobilisation System

Initial exploration used dopamine-functionalised resins with boronic acid-modified enzymes. Despite successful immobilisation, progressive dopamine oxidation caused resin darkening and compromised stability during storage.

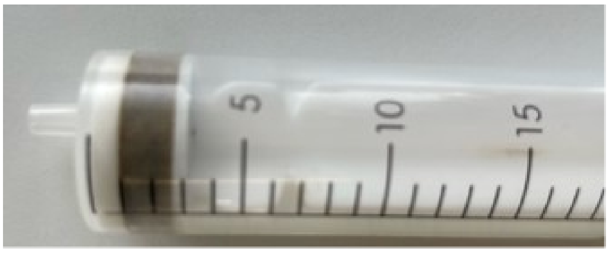

Figure S1: Darkening of dopamine-functionalised resin over time due to catechol oxidation, highlighting the chemical instability that prompted the inverted design strategy.

Enzyme cleavage under initial design conditions

Enzyme cleavage monitoring proved challenging due to protein precipitation under acidic desorption conditions, limiting quantitative assessment of regeneration efficiency.

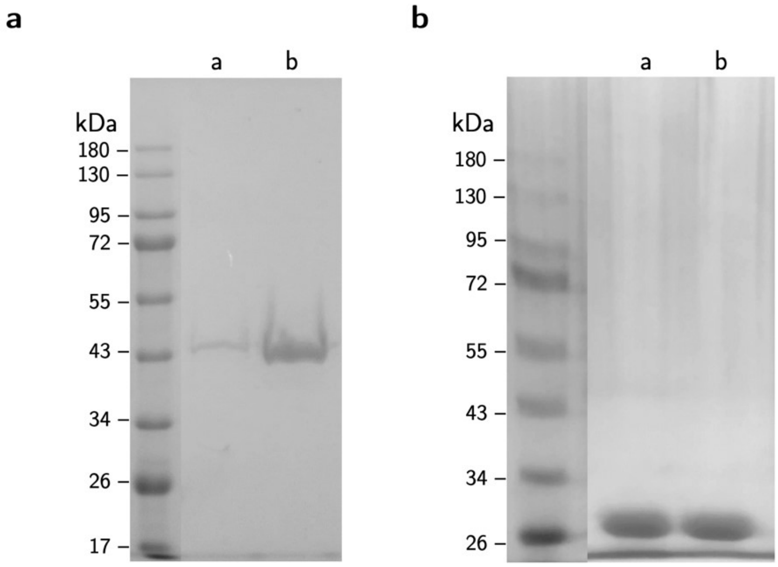

Figure S2: SDS-PAGE gel of solubilised protein in a solution of 8 M urea. **a** Incubation of boronic acid-functionalised *Cb*FDH immobilised on catechol-functionalised methacrylate in 0.1 M H_2SO_4 followed by incubation in 8 M urea (a), incubation of boronic acid functionalised *Cb*FDH in 8 M urea (b). **b** Incubation of boronic acid-functionalised *Ms*AcT immobilised in catechol-functionalised agarose in 0.1 M H_2SO_4 followed by incubation in 8 M urea (a), incubation of boronic acid-functionalised *Ms*AcT in 8 M urea (b).

Reversible Immobilisation and Resin Regeneration with Alizarin-Labelled BSA

Alizarin-labelled BSA was used to assess the specificity and reversibility of boronate ester formation on boronic acid agarose. Upon incubation at pH 8, the red protein solution rapidly decoloured as the resin turned orange, confirming binding via boronate ester formation. The immobilised protein remained attached during washing and resisted thermal desorption, but was completely released under acidic conditions (70% ethanol/30% water, pH ≈ 3), regenerating the resin to its original white state. Quantitative analysis of flow-through, boiling, and cleavage fractions over five consecutive cycles using A_{520} and BCA assays confirmed reproducible binding, stability, and full release after each regeneration.

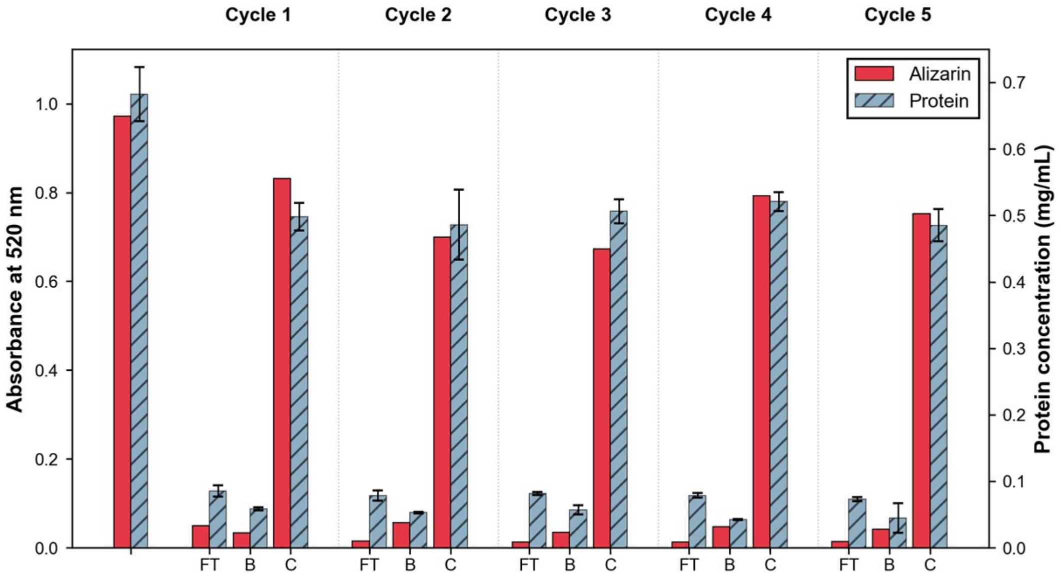

Figure S3: Tracking of alizarin-labelled enzyme over five load-cleave cycles. Fractions: **FT** (flow-through), **B** (boiling test), **C** (acidic cleavage). Bars report alizarin A_{520} (red, left y-axis) and [protein] by BCA assay (blue, right y-axis).

Operational Stability

A. Assays pH Stability: To assess the stability of boronate ester bonds across the pH scale, 50 mg of boronic acid-functionalised resin was first loaded with 1 mL of 1 mM alizarin solution at pH 8 for 2 h. After separation and washing, the alizarin-loaded resins were incubated with 1 mL of 100 mM buffer at pH values ranging from 3 to 10 for 2 h at room temperature with gentle shaking. After incubation, the resin was separated by centrifugation and the flow-through was collected. From each sample, 100 µL was neutralised by addition of 100 µL of 1 M potassium phosphate buffer pH 8, and absorbance at 520 nm was measured to quantify alizarin release during the 2 h incubation period.

B. Buffer Compatibility: To assess stability against competing nucleophiles and additives, 50 mg of boronic acid-functionalised resin was loaded with alizarin-labelled BSA (5 mg/g loading) following the standard immobilisation protocol. After washing, the immobilised enzyme was incubated with 1 mL of the following solutions for 2 h at room temperature with gentle shaking: 100 mM HEPES pH 7.5, 100 mM TRIS pH 8.0 (supplemented with 300 mM NaCl), 100 mM KPi pH 8.0 (supplemented with 1 mM DTT), 100 mM KPi pH 8.0 (supplemented with 10% w/w glycerol), and 250 mM sodium formate. After incubation, the resin was separated by centrifugation, and the amount of leached protein in the supernatant was quantified by Bradford assay.

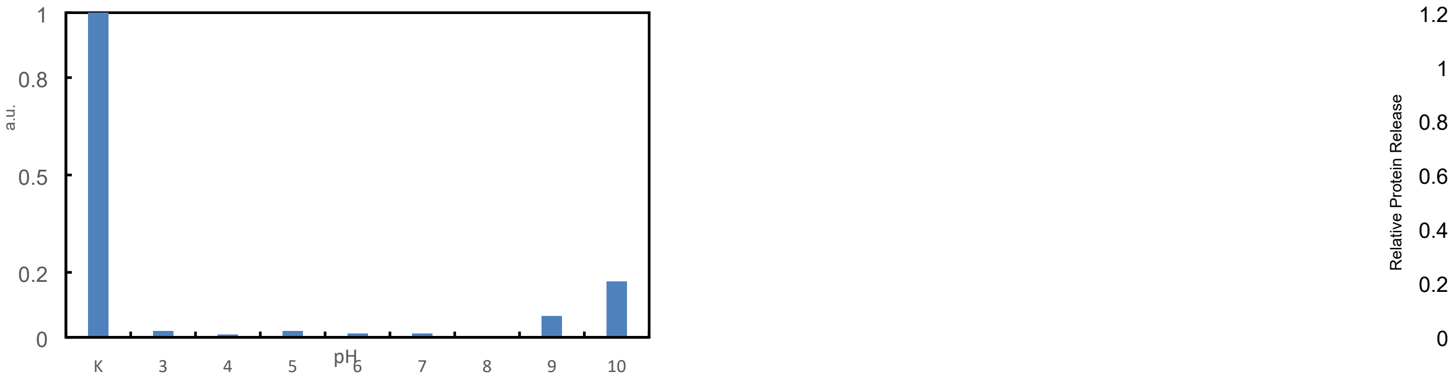

Figure S4: A. pH stability of immobilised alizarin on boronic acid resin. Alizarin remains stably bound across pH 3-8, with minimal release at pH 9-10 (~6-17%). “K” denotes the control sample (alizarin solution before loading on the resin). **B.** The immobilised enzyme was incubated for 2 h in the presence of high ionic strength (TRIS + 300 mM NaCl), reducing agents (DTT), competing polyols (10% Glycerol), and substrates (Formate). Protein leaching was negligible (<6%) in all cases as determined by Bradford assay of the supernatant relative to the initial load control ("K"), confirming the robustness of the linkage against common biocatalytic additives.

Determination of Degree of Labelling (DoL)

Due to light scattering from the protein, a baseline shift was often observed in the UV-Vis spectra, rendering single-point absorbance measurements at 520 nm inaccurate. To correct for this, the alizarin content was quantified by integrating the peak area between 410 and 650 nm. A calibration curve relating integrated area to concentration was established using free alizarin-IDA (y = 538.88x, $R^2 = 0.999$), allowing precise quantification of the attached dye regardless of baseline effects.

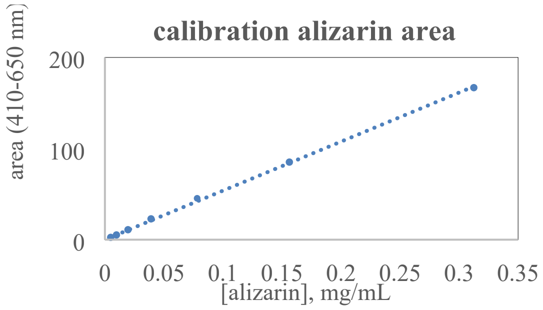

Figure S5: Calibration curve of free alizarin-IDA in 50 mM KPi pH 8.0 ($y = 538.88x$, $R^2 = 0.999$). To correct for baseline shifts caused by protein light scattering, the alizarin content was quantified by integrating the absorbance peak area between 410 and 650 nm rather than relying on single-point absorbance at 520 nm.

Immobilisation Kinetics

Kinetic experiments were performed using *Ts*RTA as a model enzyme. To ensure the alizarin signal remained above the detection limit of the spectrophotometer during the time-course, the enzyme loading was increased to 20 mg protein per gram of resin (using 50 mg resin in 1 mL volume), deviating from the standard 5 mg/g protocol. The resin was incubated with the enzyme solution with gentle agitation. At specified time intervals (0, 0.5, 2, 4, 6, 8, 10, 12 min), the suspension was briefly settled, and supernatant samples were withdrawn. The immobilisation yield (IY%) was calculated by measuring the residual protein concentration in the supernatant using both the Bradford assay and alizarin absorbance at 520 nm. Control experiments were performed identically using unfunctionalised agarose beads.

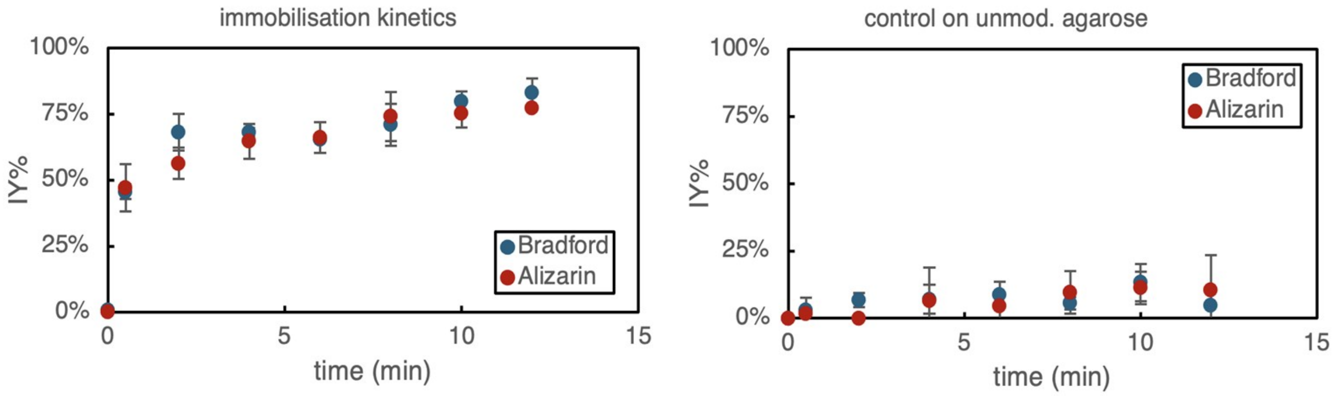

Figure S6: Immobilisation kinetics of alizarin-labelled *Ts*RTA. (Left) Time course of immobilisation on boronic acid-functionalised agarose. The process is rapid, with ~50% binding occurring within the first minute and >75% achieved within 12 minutes. The strong correlation between Bradford assay (blue) and alizarin absorbance (red) validates the use of the dye as a visual tracker for the protein. (Right) Control experiment on unmodified agarose showing minimal non-specific adsorption (<15%) over the same time frame. Error bars represent standard deviation of triplicates. Note: Experiments were performed at 20 mg/g loading to ensure detection sensitivity.

Experimental

Protein Expression

All enzyme genes were cloned into pET-28b(+) vectors using standard molecular biology techniques. Plasmids were transformed into *E. coli* BL21(DE3) cells and expressed under optimised conditions. Details for each enzyme are provided in **Table S1**.

Protein Purification

All His_6-tagged enzymes were purified by Ni-NTA affinity chromatography. Cells were lysed by sonication (40% amplitude, 5 s on/10 s off pulses, 8-12 min on ice), debris removed by centrifugation (14,500 rpm, 45 min, 4 °C), and clarified lysates applied to Ni-NTA resin either using an ÄKTA pure FPLC system or by batch/gravity flow methods. Purification buffers were optimised for each enzyme regarding buffer composition pH (7.0-8.0), salt concentration (0-300 mM NaCl), and imidazole concentrations (10-30 mM binding, 300 mM elution). Detailed purification conditions for each enzyme are provided in **Table S1**. Protein concentrations were determined by measuring absorbance at 280 nm using extinction coefficients calculated by ExPASy ProtParam. For concentrations below 1 mg/mL, the Bradford assay was used with BSA as standard. Protein purity was analysed by SDS-PAGE. Purified enzymes were either aliquoted, flash-frozen in liquid nitrogen, and stored at -80 °C or stored at 4 °C until further use.

Enzyme Activity Assays

The following activity assays were used to determine enzyme activities for native enzymes, alizarin-IDA-functionalised enzymes, and immobilised enzymes. The protocols were adapted from previously reported publications. *Ms*AcT activity was determined using the p-nitrophenyl acetate (*p*NPA) hydrolysis assay. Reactions contained 1 mM *p*NPA in 100 mM potassium phosphate buffer pH 8 in 96-well plates (200 µL total volume). Reactions were initiated by addition of 10 µL enzyme solution, and absorbance at 400 nm was monitored for 2 min at 25 °C. One unit (U) was defined as the amount of enzyme producing 1 µmol p-nitrophenol per minute (ε = 15.0 mM^{-1} cm^{-1}).[1]
*Cb*FDH activity was measured by monitoring NADH formation at 340 nm. The reaction mixture (200 µL) contained 100 mM sodium formate, 2 mM NAD^+ in 100 mM potassium phosphate buffer pH 7.5 at 25 °C. One unit was defined as the amount of enzyme producing 1 µmol NADH per minute (ε = 6.22 mM^{-1} cm^{-1}).[2]

*Ts*RTA activity was assessed by monitoring acetophenone formation at 245 nm (ε = 12.6 mM^{-1} cm^{-1}). Reactions contained 2.5 mM pyruvate, 2.5 mM (*R*)-methylbenzylamine, 0.25% DMSO in 50 mM potassium phosphate buffer pH 8.0 at 25 °C. One unit was defined as the formation of 1 µmol acetophenone per minute.[3]
*Sp*LCD activity was determined in 200 µL reactions containing 10 mM L-lysine in 100 mM potassium phosphate buffer pH 7.0 at 37 °C with 150 rpm shaking. Reactions were initiated with 1 mg/mL enzyme. L-pipecolic acid formation was monitored by HPLC after FMOC derivatisation: 50 µL sample was mixed with 100 µL of 100 mM borate buffer pH 9.0 and 200 µL of 15 mM FMOC-Cl in acetonitrile. One unit was defined as the amount of enzyme producing 1 µmol L-pipecolic acid per minute.[4]
All activity assays were performed in triplicate. For immobilised enzyme activity measurements, assays were conducted using two ways depending on enzyme activity levels. For enzymes with moderate activity (*Sp*LCD, *Cb*FDH), 50 mg of resin was loaded into Mini Bio-Spin chromatography columns (Bio-Rad, 1.2 mL bed volume) and incubated with 1 mL reaction mixture at appropriate temperature with gentle rotation. For high-activity enzymes (*Ms*AcT, *Ts*RTA), reactions were performed in 5 mL polypropylene syringes equipped with polyethylene frits, using 50 mg resin in 5 mL reaction volume to prevent fast substrate depletion. At specified time points, the reaction mixture was separated from the resin by brief centrifugation (Bio-Spin columns, 1 min at 1000 × g) or gravity filtration (syringe format). The collected supernatant was immediately analysed as described above. Immobilised activities were expressed as U/g support, with recovered activity (RA) calculated as the percentage of specific activity retained after immobilisation compared to the corresponding unmodified free enzyme.

Resin Preparation and Functionalisation

Epoxy activation of agarose. Agarose 6BCL beads (1 g) were suspended in a solution of 4.4 mL distilled water and 1.6 mL acetone on ice with gentle agitation. NaOH (338 mg) and $NaBH_4$ (40 mg) were added sequentially with stirring. Epichlorohydrin (1.1 mL) was added dropwise on ice under continuous gentle agitation. After overnight incubation at room temperature, the resin was filtered and washed with distilled water until neutral pH was achieved.
Aldehyde functionalisation. Both epoxy-agarose and EP400 epoxy resin (Resindion S.R.L.) were converted to aldehyde-functionalised supports. For each gram of resin, 10 mL of 100 mM H_2SO_4 was added and incubated overnight at room temperature with gentle shaking to hydrolyse epoxides to vicinal diols. After washing with distilled water (5 × 10 mL), resins were suspended in 10 mL of 30 mM $NaIO_4$ and incubated for 2 h at room temperature to oxidise vicinal diols to aldehydes. Resins were washed extensively with distilled water.
Boronic acid functionalisation. Glyoxyl resins (1 g) was suspended in 10 mL of 100 mM sodium carbonate buffer pH 10 containing 50 mM 3-aminophenylboronic acid. After 2 h incubation at room temperature, sodium borohydride was added to a final concentration of 10 mM and incubated for an additional 2 h at 4 °C to reduce Schiff bases and remaining aldehydes. The functionalised resins were washed sequentially with 100 mM sodium phosphate buffer pH 8.0 (3 × 10 mL), 0.5 M NaCl (3 × 10 mL), and water (5 × 10 mL), then stored in 20% ethanol at 4 °C.
Dopamine functionalisation (for initial screening) was performed identically, substituting 5-fold molar excess of dopamine for 3-aminophenylboronic acid in the coupling step.

Enzyme Functionalisation

Alizarin-IDA activation and enzyme labelling. For the universal labelling protocol, enzyme concentrations were calculated based on molecular weights determined by ExPASy ProtParam. Alizarin-3-methyliminodiacetic acid (Alizarin-IDA) was activated immediately before use: 100 mM alizarin-IDA was dissolved in MES buffer pH 6.0 containing 400 mM 1-Ethyl-3-(3-dimethylaminopropyl)carbodiimid (EDC) and 400 mM N-Hydroxysulfosuccinimide (sulfo-NHS). The activation mixture was incubated for 15 min at room temperature with gentle shaking.
The pH was then adjusted to 8.0 with 1 M NaOH, and enzyme solution was immediately added to achieve a final molar ratio of 1:100 (enzyme:alizarin-IDA). The pH of the reaction was verified and adjusted, if necessary, to maintain pH 8.0. The labelling reaction proceeded for 2 h at room temperature with gentle rotation.
Following incubation, 1 mL of the reaction mixture was loaded onto a PD-10 desalting column (GE Healthcare) pre-equilibrated with 50 mM potassium phosphate buffer pH 8. The column was eluted with the same buffer, collecting 0.5 mL fractions. After the void volume, the enzyme eluted in fractions 3-5 as a characteristic pink-red solution, followed by several colourless fractions, and finally the excess unreacted alizarin-IDA eluted as a dark red band in later fractions. The pink-red enzyme fractions were pooled and stored at 4 °C. Labelling efficiency was confirmed as described in the **Determination of Degree of Labelling** section and enzyme activity immediately determined as described above.

Determination of Degree of Labelling (DoL)
To determine the number of alizarin labels per enzyme molecule, a calibration curve was first established using free alizarin-IDA. A stock solution was serially diluted (0.005–0.3 mg/mL) in 50 mM potassium phosphate buffer pH 8.0, and the absorbance was measured. The integrated peak area (410–650 nm) was plotted against concentration to generate a linear calibration curve ($y = 538.88x$, $R^2 = 0.999$). Purified alizarin-labelled enzymes were diluted to measurable concentrations, and their integrated

absorbance (410–650 nm) was recorded. The molar concentration of alizarin was calculated using the calibration curve and divided by the molar concentration of the enzyme (determined by Bradford assay) to yield the Degree of Labelling (DoL).

Immobilisation Procedures

Boronic acid density determination. To quantify boronic acid functionalisation, 50 mg of derivatised resin and control resin were loaded into separate columns and equilibrated with buffer. Alizarin solution (1.0 mL of 1 mM in 100 mM sodium phosphate pH 8.0) was added and incubated for 30 min. Bound alizarin was calculated from A_{520} measurements of input and flow-through solutions.

BSA immobilisation. Initial validation was performed using alizarin-IDA-labelled bovine serum albumin (BSA). BSA was functionalised using the universal labelling protocol described above. Boronic acid resin (50 mg) was loaded with 5 mg/g of labelled BSA following the enzyme immobilisation protocol. Binding specificity was confirmed by comparing to non-functionalised control resin. Complete protein capture was verified by BCA assay and A_{520} measurement of the flow-through. To confirm covalent boronate ester formation, the BSA-loaded resin was subjected to boiling test: 1 mL of 100 mM sodium phosphate pH 8.0 buffer was added and the suspension was incubated at 100°C for 10 min. The supernatant was collected and analysed for content of BSA. For control experiments with unlabelled BSA, boiling resulted in complete protein release, while alizarin-BSA remained bound with the resin retaining its orange colour. For cleavage experiments, the resin was treated with 3 × 1 mL of 70% ethanol/30% water with HCl, pH ~3. Each fraction was vigorously mixed and immediately eluted. Due to ethanol interference with Bradford assay and alizarin absorption at 280 nm, all fractions (initial flow-through, wash fractions, boiling test supernatant, and acidic elution fractions) were previously neutralised with 1 M potassium phosphate buffer and subsequently analysed using the Pierce BCA Protein Assay Kit for protein quantification and A_{520} for alizarin content. Complete protein removal was confirmed when both measurements returned to baseline. The regenerated resin was washed with 5 × 1 mL water followed by 3 × 1 mL buffer pH 8.0 and reloaded with fresh alizarin-BSA for 5 complete cycles.

Reusability Studies

Extended cycling experiments. Resin reusability was evaluated using *Ms*AcT as a representative biocatalyst. Each complete cycle consisted of three phases: (i) enzyme loading, (ii) repeated catalytic use, and (iii) enzyme removal and resin regeneration. For enzyme loading, 50 mg boronic acid resin was loaded with 5 mg/g alizarin-functionalised *Ms*AcT as described above. The immobilised enzyme was then subjected to 10 consecutive catalytic reactions: 1 mM *p*NPA in 100 mM potassium phosphate buffer pH 8.0 for 10 min at 25 °C, with activity measured after cycles 1, 5, and 10. Between reactions, the resin was washed with 3 × 1 mL buffer.

After 10 catalytic uses, the spent enzyme was removed by acidic cleavage as described above. Complete enzyme removal was confirmed by the colour transition from orange to white resin. The regenerated resin was washed with 5 × 1 mL water followed by 3 × 1 mL buffer pH 8.0. This complete sequence (loading → 10 uses → regeneration) was repeated 5 times. Fresh alizarin-*Ms*AcT was loaded for each cycle, and activity was measured to confirm consistent resin performance.

Immobilisation Kinetics

Kinetic experiments were performed using *Ts*RTA as a model enzyme. To ensure the alizarin signal remained above the detection limit of the spectrophotometer during the time-course, the enzyme loading was increased to 20 mg protein per gram of resin (using 50 mg resin in 1 mL volume), deviating from the standard 5 mg/g protocol. The resin was incubated with the enzyme solution with gentle agitation. At specified time intervals (0, 0.5, 2, 4, 6, 8, 10, 12 min), the suspension was briefly settled, and supernatant samples were withdrawn. The immobilisation yield (IY%) was calculated by measuring the residual protein concentration in the supernatant using both the Bradford assay and alizarin absorbance at 520 nm. Control experiments were performed identically using unfunctionalised agarose beads.

Analytical Methods

UV-Vis spectroscopy. Alizarin-IDA labelling was monitored at 520 nm using a BioTek Microplate Reader spectrophotometer. Spectra were recorded from 400-700 nm in 50 mM potassium phosphate buffer pH 7.5.

Protein quantification. Protein concentrations were determined by Bradford assay (Sigma-Aldrich) using BSA as standard, except for ethanol-containing samples where Pierce BCA Protein Assay Kit (Thermo Fisher Scientific) was used according to manufacturer's instructions. For purified proteins >1 mg/mL, absorbance at 280 nm was measured using extinction coefficients calculated by ExPASy ProtParam.

HPLC analysis. For *Sp*LCD activity assays, amino acid products were derivatised with FMOC-Cl and analysed on a Dionex UltiMate 3000 system equipped with a C18 column (3.5 µm, 2.1 × 100 mm, Waters). Gradient elution: 5:95 to 95:5 water/acetonitrile containing 0.1% TFA over 4 min at 0.8 mL/min, 45 °C. Detection at 265 nm (Lys-FMOC: 7.12 min, L-PA-FMOC: 6.81 min).

References

1 M. L. Contente, S. Farris, L. Tamborini, F. Molinari and F. Paradisi, *Green Chem.*, 2019, **21**, 3263–3266.
2 V. Marchini, A. I. Benítez-Mateos, D. Roura Padrosa and F. Paradisi, *Front. Catal.*, DOI:10.3389/fctls.2021.790461.
3 P. Díaz-Kruik, D. Roura Padrosa, E. Hegarty, H. Lehmann, R. Snajdrova and F. Paradisi, *Org. Process Res. Dev.*, 2024, **28**, 2683–2691.
4 K. Stalder, A. I. Benítez-Mateos and F. Paradisi, *ChemCatChem*, 2024, **16**, e202301671.
